# Smoking behavior of males attending the quit tobacco clinics in Bahrain and their knowledge on tobacco smoking health hazards

**DOI:** 10.1186/s12889-018-5104-7

**Published:** 2018-01-30

**Authors:** Randah R. Hamadeh, Jamil Ahmed, Maha Al Kawari, Sharifa Bucheeri

**Affiliations:** 1 Department of Family and Community Medicine, College of Medicine and Medical Sciences, Arabian Gulf University, P.O. Box 26671, Manama, Kingdom of Bahrain; 2 National Health Regulatory Authority, P.O. Box 11464, Manama, Kingdom of Bahrain

**Keywords:** Waterpipe tobacco smoking, Cigarette smoking, Tobacco, Cessation, Smoking behavior, Secondhand smoking, Knowledge

## Abstract

**Background:**

One third of Bahraini adult (20–64 years) males and 7.0% of females use some form of tobacco. The corresponding rates for cigarette and waterpipe tobacco smoking (WTS) are 11.0% and 6.0%, respectively. The objective of the study was to determine the knowledge on tobacco smoking and past smoking related behavior of male patients attending the Quit Tobacco Clinics (QTC) in Bahrain.

**Methods:**

A sample of 339 male clinic attendees was taken proportional to the population distribution in the three QTC at Al Hoora Health Center, Hamad Kanoo Health Center, and Bank of Bahrain and Kuwait Health Center. Data collection was performed until the sample size was completed (September 2015 to December 2016). Knowledge on the health effects of cigarette and WTS was examined based on 10 statements on cigarette and similar ones on WTS. Respondents “agreeing” with the statements were considered knowledgeable and those “disagreeing” or responding “don’t know”, not knowledgeable. All the “agree” responses for cigarette/WTS were summed across the 10 health effects and average health knowledge scores for cigarette/WTS were computed.

**Results:**

Most of the study participants were Bahraini nationals, ever married and educated with at least secondary level. The majority (65.8%) of participants smoked a single type of tobacco product, and the rest, two (28.0%) or three or more (6.2%). Age of starting cigarette and WTS was 16.2 ± 4.0 and 19.3 ± 6.7 years, respectively. The majority (81%) smoked in the presence of other family members and 26.3% in the presence of a child. 76.2% smoked in the presence of others in their cars. 18.9% of the attendees had quit smoking at the time of interview. 81% of the participants knew about the hazards of both cigarette and WTS with a significantly higher (*p* = 0.0001) mean knowledge score for cigarette (93.3 ± 3.0%) than WTS (85.2 ± 2.1%).

**Conclusion:**

The relative lack of knowledge on the hazards of WTS in a sample of Arab country population with an increasing trend of WTS warrants the attention of health policy makers in the country and region.

## Background

Despite the fact that tobacco prevalence has declined in many countries, about 22% of the global population still smokes any form of tobacco and six million people die annually from tobacco related diseases [1]. Successful tobacco use treatment can revert health status to normal. The risk of premature death and morbidity are reduced by 90% if smokers quit before the age of 30 years or by half if they quit at the age of 50 years [2]. Appropriate tobacco treatment services including national quit lines and nicotine replacement therapy (NRT) are available as government health plan covered services in most developed countries. National quit line, and both NRT and some tobacco treatment services are cost-covered in thirteen developed, eight middle and seven low-income countries. However, since 2010, the progress has been very slow; as only four additional countries have signed up to include cost covered tobacco treatment services [3].

The tobacco smoking rates are still high in countries of the Eastern Mediterranean Region which include the Gulf Cooperation Council (GCC) countries despite the serious antismoking efforts including the establishment of tobacco treatment services [4, 5]. In some countries of the region, the age standardized prevalence rates among men are very high as in Jordan and Tunisia where the rates are 43% and 45%, respectively [2]. About a fifth of the population of GCC countries smoke cigarettes and WTS is a growing concern as the prevalence is on the increase [4].

Bahrain imposes a complete ban on tobacco advertising, promotion and sponsorship [6]. Despite of this, the non-communicable diseases (NCDs) survey in 2007 showed that 20% of Bahrainis of both sexes aged 20 to 64 years “currently” smoked any form of tobacco product at the time of survey. Males smoked at considerably higher rates (33.4%) than females (7.1%) any type of tobacco product. Further, significant proportions of males (11%) and females (6%) were current *waterpipe* smokers then [7]. A recent study on primary healthcare physicians in Bahrain reported that 11% of the physicians ever smoked and 8.6% were smokers at the time of survey [8]. The prevalence of smoking of all types of tobacco among male medical students in Bahrain increased from 27.5% in 1993 to 35.2% in 2005. The corresponding rates for females increased from 2.3% to 7.0%, respectively [9].

About 84% of patients can quit tobacco smoking if they receive intensive health education advice from a physician [1]. Tobacco smoking in any form can lead to chronic addiction and quitting smoking is often found to be difficult for smokers without assistance [4, 10]. Dedicated tobacco treatment clinics are necessary for communities to receive support in quitting smoking and therefore reduce the burden of NCDs. Generally, physicians have limited available time to counsel or support patients to quit smoking. One study from the region reported that only 12% of dentists counseled their patients against smoking [11].

The burden of lung cancer and cardiovascular diseases continue to remain high in Bahrain. The mortality rates for circulatory diseases and neoplasms are as high as 108.4 and 55.6 per 100,000 among nationals [12]. Lung cancer is the most common cancer among Bahraini males and the second among Bahraini females with annual age standardized incidence rates of 26.1/100, 000 and 10.0/100, 000, respectively [13]. Furthermore, Bahrain has the highest lung cancer incidence rate among GCC countries [14].Similarly, circulatory diseases accounted for 36.6% of 2860 total deaths reported in 2015 in Bahrain [12]. Since the Bahraini health system works under limited resources, tobacco control and prevention would significantly contribute in alleviating the health burden imposed by lung cancer, cardiovascular diseases and the many other conditions associated with tobacco use. Bahrain ratified the World Health Organization’s Framework Convention on Tobacco Control in 2004 and has passed an antismoking law in 1994 and a modified one in 2009 [15, 16]. Quit Tobacco Clinics (QTC) were established in Bahrain since 2004 in governmental primary healthcare centers with the first clinic in Al Hoora Health Center (AHHC). It was followed by two more clinics in two health centers (Hamad Kanoo Health Center (HKHC) in 2012, and Bank of Bahrain and Kuwait Health Center (BBKHC), in 2014). More details on these clinics is provided in an earlier publication which examined smoking cessation rates and satisfaction of the attendees of these clinics [5].

The available literature in the region mainly focuses on the burden and trends rather than the factors related to tobacco use and treatment. The objective of this study was to determine the behavior and knowledge about tobacco use and past smoking related behavior of male attendees of QTC in Bahrain. We are hopeful to provide decision makers involved in tobacco control in Bahrain further understanding of the tobacco users to adopt more comprehensive strategies in tobacco prevention.

## Methods

### Study population

The study population included all male smokers attending the QTC at AHHC, HKHC and BBKHC where, respectively 641, 175 and 63 patients attended the clinics in 2014. A sample of 339 male clinic attendees was taken stratified according to the QTC population in the three health centers during 2014. Females were excluded from the study as they made up only a very small proportion of the QTC attendees. Participants were selected consecutively based on their written consent to participate in the study. Data collection started in September 2015 and continued until the sample size was completed in December 2016.

The data were collected through interviews based on a structured questionnaire in both Arabic and English languages. Three trained data collectors conducted the face to face interviews. The questionnaire included sociodemographic characteristics, history of smoking including quit attempts, knowledge on the hazards of all types of tobacco and attitude towards tobacco smoking. The data was analyzed through the Statistical Package for the Social Sciences version 20. Descriptive analysis, T-Test and ANOVA for quantitative continuous variables and Chi-Square test for quantitative categorical variables were performed.

Cigarette smokers were classified into light (1–10 cigarettes daily), moderate (11–20 cigarettes daily) and heavy (> 20 cigarettes daily) smokers. Knowledge on the health effects of cigarette and WTS was examined based on 10 statements on cigarette and similar ones on WTS. The participants either “agreed”, “disagreed” or “did not know” that cigarette/WTS causes lung, mouth, laryngeal and esophageal cancer; heart disease, stroke, triggered an attack among asthmatics, can cause chronic respiratory illness and is harmful to health.

Respondents “agreeing” with the statements were considered knowledgeable and those “disagreeing” or responding “don’t know”, not knowledgeable. All the “agree” responses for cigarette/WTS were summed across the 10 health effects and average health knowledge scores for cigarette/WTS were computed.

Ethical approval was obtained from the Research and Ethics Committee of the College of Medicine and Medical Sciences, Arabian Gulf University and from the Research, Technical and Support Committee, Ministry of Health, Bahrain. Further, a written consent was sought from the participants of the study.

## Results

The mean age of the participants was 33.4 ± 12.9 years with about one third, 25 years of age or younger. Almost half had completed up to secondary level of education, 64.6% were ever married, three fourths were Bahraini nationals and a quarter earned ≥ 900 Bahraini Dinar (BHD) (1 BHD = 2.65 US Dollar) a month (Table 1).

**Table 1:** Sociodemographic characteristics of study participants

	Categories	Number	Percent
Age (Years)	≤ 25	104	31.1
	26–40	131	39.2
	41–55	82	24.6
	56–70	17	5.1
	Total	334	100
Educational Level	Primary	27	8.0
	Intermediate	71	21.1
	Secondary	154	45.7
	Graduate	85	25.3
	Total	337	100
Marital status	Never Married	120	35.4
	Ever Married	219	64.6
	Total	339	100
Nationality	Bahraini	257	75.8
	Other Arab	65	19.2
	Other	17	5.0
	Total	339	100
Income (BHD)	< 300	13	6.8
	300–599	68	35.6
	600–899	61	31.9
	≥900	49	25.7
	Total	191	100

### Smoking behavior

**Fig. 1:**
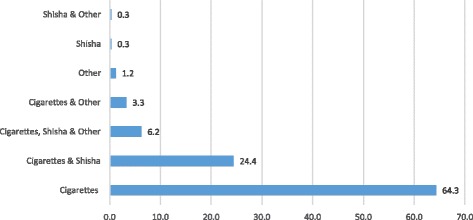
Type of tobacco smoked

The majority (65.8%) of participants smoked a single type of tobacco product, and the rest, two (28.0%) or three or more (6.2%) (Fig. 1). The commonest type smoked was cigarettes (98.2%), followed by *shisha* (31.3%), *midwakh* (4.5%), pipe (3.0%), cigar (2.7%) and cheroot (1.8%). Age of starting cigarette and WTS were 16.2 ± 4.0 and 19.3 ± 6.7 years, respectively. Cigarette smokers smoked 25.1 ± 14.0 cigarettes daily and waterpipe smokers, 15.5 ± 15.0 times in a typical week. Marlboro, Davidoff and LM were the top three brands of cigarettes smoked and *Fakher* was the commonest waterpipe brand (76.7%). Friends was the most stated main reason for starting cigarette (33.7%) and WTS (46.5%).

Eighty six percent smoked their first cigarette of the day within an hour after waking up, of whom, 36.7% smoked it within 5 minutes (Table 2). Men aged 26–40 years were more likely to be moderate and heavy cigarette smokers as compared to the younger and older age groups (p = < 0.001). Further, there were no significant differences in the age starting to smoke cigarettes among the three types of smokers. Similarly, the ever married smoked a higher number of cigarettes than the never married (*p* < 0.001). There were no significant differences in WTS levels and sociodemographic variables. However, waterpipe smokers with a duration of smoking more than 10 years were mostly middle aged and married (*p* < 0.05). Table 3 shows the duration of cigarette smoking with the sociodemographic variables. We found that more of those aged between 41 and 55 years (*p* < 0.0001) and ever married (*p* < 0.001), smoked longer than others and those with less than 300 BHD income a month smoked for more than 10 years (*P* < 0.05) (Table 3).

**Table 2:** Cigarette smoking behavior of the participants at the QTC

	Categories	Number	Percent
Brand of cigarettes mostly smoked	Dunhill	51	15.5
	Marlboro	86	26.1
	LM	58	17.6
	Davidoff	70	21.2
	Others	65	19.7
	Total	330	100
The reason to start cigarette smoking	Friends	110	33.7
	Personal Problems	21	06.4
	Imitation	20	06.1
	Experimentation	50	15.3
	Curiosity	12	3.7
	Teenage	14	4.3
	Others	99	30.4
	Total	326	100
The first cigarette smoked daily	Within 5 min	123	36.7
	6–30 min	75	22.4
	31–60 min	90	26.9
	Other	47	14.0
	Total	335	100
The reason to start waterpipe smoking	Friends	46	46.5
	Experimentation	21	21.2
	Curiosity	12	12.1
	Others	20	20.2
	Total	99	100
Brand of waterpipe mostly smoked	*Naklah*	6	10.0
	*Fakher*	46	76.7
	Mixed	3	5.0
	Don’t Know	5	8.3
	Total	60	100

**Table 3:** Type of cigarette smokers and duration of cigarette smoking by sociodemographic variables

Variable	Categories	Smoker (Cigarette) Per day			*P*-Value	Duration of Cigarette Smoked (years)		*P*-Value
		Light Smoker [1–10] *n* (%)	Moderate Smoker [11–20] *n* (%)	Heavy Smoker (> 20) n (%)		< 10 n (%)	≥ 10 n (%)	
Age (Years)	≤ 25	27 (60.0)	48 (34.8)	29 (19.2)	< 0.0001	101 (40.7)	0 (0)	< 0.0001
	26–40	13 (28.9)	58 (42.0)	60 (39.5)		120 (48.4)	7 (9.1)	
	41–55	4 (8.9)	25 (18.1)	53 (35.1)		26 (10.5)	55 (71.4)	
	56–70	1 (2.2)	7 (5.1)	9 (6.0)		1 (0.4)	15 (19.5)	
	Total	45 (100)	138 (100)	151 (100)		248 (100)	77 (100)	
Educational Level	Primary	4 (8.9)	7 (5.1)	16 (10.4)	0.844	17 (16.9)	9 (11.3)	0.660
	Intermediate	8 (17.9)	30 (21.7)	33 (21.4)		51 (20.6)	17 (21.3)	
	Secondary	21 (46.7)	67 (48.6)	66 (42.6)		117 (47.2)	34 (42.5)	
	Graduate	12 (26.7)	34 (24.6)	39 (25.2)		63 (25.4)	20 (25)	
	Total	45 (100)	138 (100)	155 (100)		248 (100)	80 (100)	
Marital Status	Never Married	31 (68.9)	52 (37.7)	37 (23.7)	< 0.0001	114 (46)	2 (2.5)	< 0.001
	Ever Married	14 (31.1)	86 (62.3)	119(76.3)		134 (54)	79 (97.5)	
	Total	45 (100)	138 (100)	156 (100)		248 (100)	81 (100)	
Nationality	Bahrain	36 (80)	108 (77.7)	113 (72.9)	0.234	186 (74.7)	63 (77.8)	0.228
	Other Arab	6 (13.3)	22 (15.8)	37 (23.9)		53 (21.3)	11 (13.6)	
	South Asian	3 (6.7)	9 (6.5)	5 (3.2)		10 (4)	7 (8.6)	
	Total	45 (100)	139 (100)	155 (100)		249 (100)	81 (100)	
Income (BHD)	< 300	3(14.3)	7 (8.8)	3(3.3)	0.557	11 (8.3)	2 (3.6)	0.534
	300–599	6 (28.6.)	28 (35)	34 (37.8)		46 (34.8)	22 (39.3)	
	600–899	6 (28.6.)	27 (33.8)	28 (31.1)		44 (33.3)	16 (28.6)	
	≥900	6 (13.3)	18 (22.5)	25 (27.8)		31 (23.5)	16 (28.6)	
	Total	21 (100)	80 (100)	155 (100)		132 (100)	56 (100)	
Number of Children	None	0 (0)	9 (10.6)	13 (11.4)	0.075	18 (13.7)	4 (5.2)	0.471
	1–2	9 (64.3)	32 (37.6)	30 (26.3)		60 (45.8)	10 (13)	
	3–4	5 (35.7)	30 (35.3)	47 (41.2)		42 (32.)	37 (48.1)	
	5–6	0 (0)	14 (16.5)	24 (21.1)		11 (8.4)	26 (33.8)	
	Total	14 (100)	85 (100)	114 (100)		131 (100)	77 (100)	

**Fig. 2:**
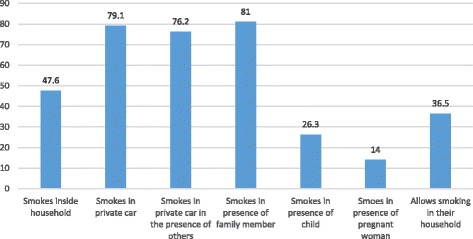
Smoking behavior

Over half (57.6%) of the participants had other family members who smoked at the household of whom fathers (22.2%), brothers (39.2%) and both fathers and brothers (11.9%) constituting the majority (73.3%). About half (47.6%) of the participants smoked inside their home mostly in the bedroom (28.8%).Other places included all rooms (13.1%), sitting room (20.6%), bathroom (11.9%), kitchen (5.0%) and the rest (20.6%) in other rooms. 81% smoked in the presence of other family members at home and 26.3% in the presence of a child. 76.2% smoked in the presence of others in their car (Fig. 2).

### Tobacco quitting behavior

At the time of interview, 18.9% of the attendees had quit smoking with a higher proportion among the Bahraini (21.7%) than the non-Bahraini (9.0%) (*p* = 0.012). Of those who quit, 3.2% had quit for 6 months or more, 73.4% for ≤ 30 days and the rest (23.4%) for over a month but less than 6 months. More participants in the older age groups (> 40 years) had tried to quit smoking than the younger ones (≤ 40 years). Of those who were still smoking at the time of interview, 54.4% were very motivated to quit, 43.0% somehow motivated and 2.7% not motivated. Those with lower income (< 300 BHD) were less motivated (45.7%) than those with higher (67.5%) income (*p* = 0.001). Health was the main reason for considering quitting smoking in three quarters of the clinic attendees. Among mixed smokers of both cigarettes and *shisha*, the former was the main type of tobacco they wished to quit.

### Tobacco related knowledge

The percentage of participants who agreed with the knowledge statements was high (≥ 81.7%) for both cigarettes and *shisha* with higher proportions to all the cigarettes’ knowledge statements compared to those of *shisha* (Table 4). The statement “Cigarette smoking can cause cancer of the esophagus” ranked the lowest (88.2%) for cigarettes whereas “*Shisha* smoking can cause stroke” was for *shisha* (81.7%). The mean knowledge score was significantly higher (*p* = 0.0001) for cigarettes (93.3 ± 3%) than *shisha* (85.2± 2%). Comparison of cigarette smokers with those who smoked *shisha* only was not possible since there was one *shisha* smoker only who smoked it without other types. When comparing the knowledge of cigarette smokers with those who smoked cigarettes and *shisha* on the hazards of cigarette and WTS, the mean knowledge scores were significantly higher for cigarettes than *shisha* for both cigarette smokers and mixed smokers who smoked both cigarettes and *shisha* (Table 5). Further analyses by age, education and income showed no significant differences in the average knowledge for both cigarettes and *shisha*.

**Table 4:** Percentage who agreed to the health knowledge statements for cigarettes and *shisha* smoking

	Cigarettes (%)	*Shisha (%)*	*P*-value
Breathing smoke from other people’s smoke is harmful	93.2	85.8	0.0028
Smoking can cause lung cancer	96.2	87.0	0.0001
Smoking can cause cancer of the mouth	93.2	84.7	0.0001
Smoking can cause cancer of the larynx	92.6	83.8	0.0008
Smoking can cause cancer of the esophagus	88.2	82.6	0.0561
Smoking can cause heart disease	95.6	86.1	0.0001
Smoking can cause stroke	88.5	81.7	0.0215
Smoking can trigger an attack among those having asthma	93.2	85.3	0.0016
Smoking can cause chronic respiratory illness	94.7	86.4	0.0004
Smoking is harmful to health	97.3	88.5	0.0001
Mean ± SD	93.3 ± 3	85.2± 2.1	0.0001

**Table 5:** Smoking behavior and knowledge about health effects of tobacco

Smoking Knowledge	N	Mean	SD	Mean Diff.	*P*-Value	95% CI	
						Lower	Upper
Cigarette							
Cigarette smokers	223	95.8	12.0	2.8	0.096	-0. 497	6.017
Cigarette & shisha smokers	101	93.1	14.5				
Shisha							
Cigarette smokers	195	87.5	11.4	5.2	0.008	1.351	8.974
Cigarette & shisha smokers	101	82.4	17.6				

## Discussion

Our results showed high tobacco dependence and a statistically significant relationship between heavy cigarette smoking (> 20 cigarettes/day), and being married and relatively older (26–40 years old), are consistent with the current literature [17]. As age of marriage has increased everywhere, the relationship of age and marital status is not surprising. The fact that consumption of tobacco gradually gives rise to tolerance [18] and with time, smokers require higher doses of nicotine to make an effect. Heavy tobacco consumption is also related to stress and depression, which are more prevalent in older populations [19]. More importantly, heavy tobacco consumption has a causal association with many cancers, especially cancer of the lung [20].

Almost half of the cigarette smokers among the study population were heavy smokers. However, unlike cigarettes, waterpipe tobacco consumption could not be accurately estimated from our study. Nevertheless, our results indicated a high consumption of waterpipe. Waterpipe smokers smoked 15 times per week (> twice daily) and almost a third of them smoked it in addition to cigarettes. Considering the rising trend of its prevalence in the country, tobacco consumption among waterpipe smokers should not be underestimated [7–9]. Further, the fact that a significant proportion of cigarette smokers were also waterpipe smokers in our study is consistent with previous studies which report that cigarette smokers are more likely to smoke the waterpipe as well [21]. Our study highlighted that most chronic (> 10 years) WTS users were married and, at the same time, in middle age which indicates that the populations in in their productive years of life may be at higher risk of hazards of WTS.

Another smoking related behavior that our study highlighted is the pattern of secondhand smoking. About 81% smoked in the presence of other relatives at home, three quarters in their cars and about half had a family member smoking in their shared places at home including bedrooms, toilets, kitchens and sitting rooms. Further, the fact that 26.3% smoked in the presence of a child is shocking. This alarming pattern of secondhand smoking not only exposes nonsmokers to the harmful effects of tobacco but also makes them vulnerable to initiation and nicotine dependence [22].

Interestingly, most of the participants had made prior quit tobacco attempts and around 60% rated themselves as highly motivated to quit but was lowest among the young and those in low income. Studies have shown that the educated and high-income smokers have a higher probability of successfully quitting tobacco [23]. Socioeconomically less affluent populations elsewhere have lower tobacco quit rates than their richer counterparts [24]. Our study is consistent with existing evidence on the motivation of the tobacco quitters and their history of quit attempts. Most tobacco users are usually motivated to quit but the decision to stop depends on several factors. One of these factors is the concern about health [25], which was found to be the main reason for three quarters of the sampled population in our study to quit. One study showed that there was a relationship between tobacco users’ motivation and the overall tobacco control efforts of the country [26]. Current evidence also suggests that high motivation is related to higher probability of quitting tobacco [27]. Therefore, in view of the high motivation and frequent quit attempts in the tobacco users we studied, there may exist sufficient opportunity for helping them quit through actively reaching out to and offering them appropriate treatments.

The knowledge of the study population about the hazards of cigarette and WTS was very high as 81.7% rightly identified the correct or incorrect statements. However, the mean knowledge scores for WTS were about 8% lower (*p* = < 0.05) compared to cigarettes, implying poorer knowledge of tobacco users about the hazards of WTS. The lack of adequate knowledge of waterpipe smokers of the hazards of WTS or their discounting attitude towards the later as compared to cigarettes in our sample is consistent with evidence from the neighboring Kingdom of Saudi Arabia [28] and elsewhere [24]. In our study, the young and those of low-income lacked adequate knowledge on the effect of smoking on health and therefore they could potentially lack sufficient knowledge and motivation to quit tobacco.

This is the second study conducted in the QTC in Bahrain and the first to examine smoking behaviors that affect nonsmokers in the country. A limitation of the study is the unexpected long period of data collection since the participants were included once they visited the three QTC. However, no major changes have taken place in the country that would affect smoking behavior and knowledge towards tobacco smoking during that period. Further, unlike cigarette consumption, we did not measure tobacco consumption among waterpipe smokers. Another limitation is that we excluded women due to their small number at the time of the study. We recommend further research on Bahraini female smokers and an in depth assessment of their tobacco use and quitting behavior because of the increasing trend of WTS among them and the cultural acceptance to their smoking.

## Conclusion

The relative lack of knowledge of on the hazards of WTS in a sample of Arab country population with an increasing trend of WTS as well as the pattern of secondhand smoking warrant attention by the policy makers in the country and region. We recommend that the Ministry of Health take further steps to assist motivated smokers to quit. Further, the private health sector, nongovernmental organizations including the Antismoking Society, the Ministries of Information and Education and other stakeholders should concert their efforts in tobacco control. Future studies on tobacco smoking in Bahrain should focus on mixed smoking pattern of both cigarettes and waterpipe as well as secondhand tobacco smoking.

## Abbreviations

AHHC: Al Hoora Health Center
BBKHC: Bank of Bahrain and Kuwait Health Center
BHD: Bahraini dinar
GCC: Gulf Cooperation Council
HKHC: Hamad Kanoo Health Center
NCDs: Non-communicable diseases
NRT: Nicotine replacement therapy
QTC: Quit tobacco clinics
WTS: Waterpipe tobacco smoking
